# Slow life history leaves endangered snake vulnerable to illegal collecting

**DOI:** 10.1038/s41598-021-84745-1

**Published:** 2021-03-08

**Authors:** Chris J. Jolly, Brenton Von Takach, Jonathan K. Webb

**Affiliations:** 1 School of Life Sciences, University of Technology Sydney, Broadway, NSW 2007 Australia; 2 School of Environmental Science, Institute for Land, Water, and Society, Charles Sturt University, Albury, NSW Australia; 3 Research Institute for the Environment and Livelihoods, Charles Darwin University, Darwin, NT 0909 Australia

**Keywords:** Ecology, Zoology, Ecology, Conservation biology

## Abstract

Global wildlife trade is a multibillion-dollar industry and a significant driver of vertebrate extinction risk. Yet, few studies have quantified the impact of wild harvesting for the illicit pet trade on populations. Long-lived species, by virtue of their slow life history characteristics, may be unable to sustain even low levels of collecting. Here, we assessed the impact of illegal collecting on populations of endangered broad-headed snakes (*Hoplocephalus bungaroides*) at gated (protected) and ungated (unprotected) sites. Because broad-headed snakes are long-lived, grow slowly and reproduce infrequently, populations are likely vulnerable to increases in adult mortality. Long-term data revealed that annual survival rates of snakes were significantly lower in the ungated population than the gated population, consistent with the hypothesis of human removal of snakes for the pet trade. Population viability analysis showed that the ungated population has a strongly negative population growth rate and is only prevented from ultimate extinction by dispersal of small numbers of individuals from the gated population. Sensitivity analyses showed that the removal of a small number of adult females was sufficient to impose negative population growth and suggests that threatened species with slow life histories are likely to be especially vulnerable to illegal collecting.

## Introduction

The global wildlife trade is a multibillion-dollar industry that is a significant driver of vertebrate extinction risk^1,2^. Annually, tens of millions of plants and animals are traded across the globe to meet the burgeoning demands of consumers^3,4^, and the profits reaped from the illegal wildlife trade make it one of the world’s leading illegitimate businesses^5,6^. Whilst much of this trade consists of wildlife products (e.g., skins, ivory, horns), another major component comprises the trafficking of wild caught animals for the exotic pet trade^7,8^. Poaching of wildlife for the pet trade is ubiquitous, and it is widely recognised as an important contributor to biodiversity loss^2,9,10^. However, its impacts on wild populations are notoriously difficult to monitor and, as a result, are egregiously understudied^8,10,11^. While the number of species pushed towards extinction by illegal poaching is increasing^9^, few studies have quantified the impact that the removal of wild-caught animals for the pet trade has on wild populations (e.g.^10–12^).

International trade in live reptiles as exotic pets is a rapidly expanding market^9,13,14^, with demand for reptiles as exotic pets contributing to local population declines^15–17^, and in some instances, has been implicated in the impending extinction of some species (e.g. ploughshare tortoise *Astrochelys yniphora*^18^; *Chelodina mccordi* and *Goniurosaurus luii*^19^). Whilst Asian, African and Latin American reptile species make up a high proportion of the international pet trade, the unique morphology, behaviour, and rarity of Australia’s endemic reptiles drives a high international market value^15,20^. Although Australia has strict biosecurity regulations (*Environmental Protection and Biodiversity Conservation Act 1999*) and most states have banned the wild collection of native wildlife for the pet trade^20,21^, considerable international demand has resulted in an unregulated and highly profitable illegal black market trade in Australian reptiles. Currently, most of the information we have on the extent of this pressure comes from seizures and the presence of Australian reptile species in overseas markets^2,15,20^. Unfortunately, because we only have a limited understanding of the population dynamics of most Australian reptiles, and the threatening processes that affect them^22,23^, there is often insufficient information to assess the effects of illegal collecting on local populations.

Although populations of some geographically widespread and ecologically flexible reptile species may be relatively unaffected by moderate levels of wild harvesting (e.g.^24^), long-lived species with slow life history traits are vulnerable to over harvesting^15,25,26^. Species at a high trophic level, low population density, small geographic range size and/or slow life history are already predisposed to increased risk of extinction^27^, and long-lived species with slow growth rates, infrequent reproduction, and small litter sizes are likely susceptible to overharvesting. Unfortunately, we know so little about the threats to most Australian reptiles^22,28,29^ that it is difficult to ascertain which species may be most vulnerable to the impacts of illegal collecting. One notable exception is the threatened broad-headed snake (*Hoplocephalus bungaroides*) (IUCN status: Vulnerable; Australian Commonwealth status: Vulnerable; NSW status: Endangered), a species endemic to the Sydney region of south-eastern Australia, which has been extensively studied over the last few decades (e.g.^28,30–33^). This spectacularly coloured venomous elapid snake is highly valued by reptile keepers^31^, and the underground trade in broad-headed snakes is thought to have contributed to its decline^34^. Unfortunately, the life history traits mentioned above may make this threatened species particularly susceptible to illegal collecting for the pet trade^31,32^.

In this study, we assessed the impact of illegal collecting on endangered broad-headed snakes at two sites in the southern part of their distribution. These sites belong to one of two isolated and deeply divergent clades present in this threatened snake species^35^. Broad-headed snakes are long-lived and produce few offspring, which develop slowly and mature relatively late in life, potentially making populations extremely vulnerable to population-level disturbances such as wildfire or illegal collecting of mature individuals^31,32,36^. Our study population comprises two sandstone plateaux, one that has locked gates on fire trails to restrict access (‘gated’), and another that has no locked gates on fire trails (‘ungated’). Since 2008, study sites on the ungated plateau have been disturbed every year by humans searching for snakes, whereas sites on the gated plateau have been disturbed less frequently^37^ (also see Supplementary Information). To assess the impact of collecting at ungated sites, we used MARK software to develop models of survival to test the hypothesis that survival rates of all age classes would be lower at ungated sites than gated sites. Next, we used population viability analysis (PVA) to assess the effect of illegal collecting on the growth rate and trajectory at the ungated site as well as at the more protected gated site. By using sensitivity analyses, we identified the parameters that explained most of the variation in population persistence. We predicted that harvesting of mature adults at ungated sites would likely have the greatest impact on modelled population persistence, resulting in a much higher probability of extinction over the coming decades.

## Materials and methods

### Study species

Broad-headed snakes are small (< 90 cm snout-vent length; SVL), live-bearing, venomous elapid snakes. They are nocturnal, and juveniles feed mostly on lizards that they ambush beneath rocks. Adults have a broader diet that includes lizards, birds, and small mammals^33,38^. In Morton National Park, the location of an isolated and deeply diverged southern clade of the species^35^, broad-headed snakes grow slowly, reaching maturity at 5–6 years^39^. The species is long lived (up to 28 years), and they have long generation lengths (10.4 years^32^). Mating occurs between autumn (March) and spring (November), females ovulate in late spring, offspring are born in March and April, and females reproduce annually to biennially^32,33^. During the cooler months (May–October), broad-headed snakes occupy the western edge of exposed sandstone plateaux where they shelter under small, exposed rocks that have exfoliated away from underlying sandstone substratum^33^. During the warmer months (November–April), broad-headed snakes leave the exposed outcrops for the shelter of old growth eucalypt forests where they use tree hollows as refugia^40^. It is during the cooler months, when the majority of snakes in the population shelter beneath small, thin, exfoliating rocks on easily accessible ridgelines, that they are most vulnerable to illegal collecting^31^.

### Field sites and methods

We studied populations of broad-headed snakes located approximately 160 km south of Sydney, New South Wales, Australia. One population was located on a sandstone plateau (roughly 20 km long by 2 km wide) inside Morton National Park (henceforth, ‘gated’). The other population was located 6 km east on a sandstone plateau (roughly 27 km long by 9 km wide) on crown land (i.e. public land without tenure; henceforth, ‘ungated’). The two populations are separated by a steep valley dissected by a river, but there is some gene flow from the gated population to the ungated population^41^. At the gated population, there are two sets of locked gates at the entry and at access points of the fire trail that traverses the plateau. These gates were installed in 2008 and have since made it more difficult for people to access and disturb rock outcrops, including reptile collectors and rock collectors. By contrast, the ungated population is easily accessible to collectors because there are no locked gates on the fire trails which crisscross the plateau. Many fire trails throughout the ungated population terminate at rock outcrops, so collectors do not have to walk far (often just metres) to find broad-headed snakes. Because previous publications detailing the specific location of these populations have led to increased habitat disturbance and collecting of snakes^31^, we opted not to include location details or maps.

Since 1992 (gated population) and 2007 (ungated population), one of us (JKW) has carried out annual mark–recapture studies. Each year during late winter and/or spring, a team of herpetologists (usually 3–5 people) surveyed four study sites at the gated population and three study sites at the ungated population. At each site, the team turned all sun-exposed rocks that could be lifted without risking a back injury. All reptiles found under rocks were identified and recorded, and any broad-headed snakes or small-eyed snakes (*Cryptophis nigrescens*) were hand captured and briefly held with thick welding gloves. When a broad-headed snake or small-eyed snake was captured, the researcher measured the SVL and tail length (with a ruler, to nearest mm), determined the sex (via tail shape), and recorded mass (with spring balance, to nearest g). We recorded the snakes’ microchip number, and if the snake was unmarked, we injected a miniature transponder (Trovan Midichip 8 mm × 1.4 mm) under the skin. During surveys, the team noted whether rocks had been disturbed by humans, and if so, the nature of the disturbance (i.e. whether rocks were overturned, displaced, or broken). Disturbed rocks were easily identified because aside from being displaced or overturned, they often had the remains of squashed invertebrates or vertebrates (lizards and frogs) beneath them. Any unmarked rocks were given a unique identification number (with a paint pen, underneath the rock) to enable us to assess long-term usage of rocks by snakes, and disturbance to rocks. After processing each snake, the rock was returned to its exact original location, and the snake was released under the rock.

All procedures were approved by the University of Technology Sydney Animal Ethics Committee and were carried out in accordance with relevant guidelines and regulations under licence from state and federal wildlife agencies.

### Impacts of illegal collection of snakes from the ungated population

Because rocks at the ungated survey sites were disturbed more frequently by humans than rocks at the gated survey sites^37^, we hypothesised that humans were removing snakes for the illegal pet trade (for additional justification see Supplementary Information). If illegal collecting has occurred, broad-headed snakes from ungated sites should have lower survival rates than snakes from gated sites. To test this hypothesis, we analysed mark–recapture data collected from 2007 to 2019 using Cormack–Jolly–Seber (CJS) models in Program MARK v9.0^42^. Because previous studies have shown that survival rates vary with age and size^32,39^, we allocated each snake to one of two size classes (sub-adults and adults, SVL > 349 mm [henceforth ‘adults’], and juveniles, SVL < 350 mm). To investigate whether survival rates differed among populations, we allocated each snake to one of two populations (gated or ungated). Thus, there were four groups in the input file: gated adults, gated juveniles, ungated adults, and ungated juveniles. Next, we ran a series of models in MARK to test the following a priori hypotheses: (1) survival rates are higher in gated than ungated sites; (2) survival rates of adults and juveniles are higher in gated than ungated sites; (3) survival rates vary through time independent of location; and (4) survival rates are constant independent of location. For these hypotheses, we ran equivalent survival models in which the probabilities of recapture were constant, time-dependent, or group-dependent. We then used the Akaike Information Criterion corrected for small sample size (AICc) to identify the most parsimonious model from the candidate model set^43^.

### Estimation of life history parameters

Life history parameters were either estimated in this study or were taken from previously published studies of these populations (Table 1). Because good estimates of parameter uncertainty are necessary to construct informative stochastic demographic models, we used program MARK to obtain estimates of environmental (process) variation around survival rates (Supplementary Information S2). We did this using the variance components subroutine in MARK (appendix D, MARK Book v 19, see^44^). For this analysis, we used the mark–recapture dataset for the gated population, with two groups (juveniles and adults). We then ran CJS models, and estimated variance components for each age class separately from the model $S (group \times time) p (constant)$. Initial population size estimates were obtained from previous analyses that extrapolated estimates from study sites to the plateau (Table 1). Although our initial population sizes are extrapolations upon estimates and are potentially imprecise, we accounted for this uncertainty by varying initial population size to see what effect it had on population growth rates (see below). Using previously published literature from these populations and parameters estimated in this study, we obtained the following life history parameters required for population viability analysis: (1) age at maturity for males and females; (2) maximum age of reproduction; (3) maximum litter size; (4) sex ratio at birth; (5) percentage of females breeding annually; (6) mean number of offspring per female; (7) biological survival rates calculated by age class; and (8) dispersal rates (Table 1).

**Table 1:** Life history parameters used in the base scenario for population viability analysis (PVA) of gated and ungated populations of broad-headed snakes in Morton National Park, New South Wales, Australia.

Parameters	Values		Source
	Gated	Ungated	
**Reproductive system**			
Age at maturity	Male—5 years	Male—5 years	Webb et al.^39^
	Female—6 years	Female—6 years	Webb et al.^39^
Maximum age of reproduction	26 years	26 years	Webb unpubl. data
Maximum litter size	9	9	^32^
Sex ratio at birth	1:1	1:1	Fitzgerald unpubl. data
**Reproductive rate**			
Percentage of adult females breeding annually (± SD)	56 ± 10%	56 ± 10%	^32^See Supplementary Information S2 and Table S1 for calculation of SD
Mean number of offspring per female (± SD)	6.8 ± 1	6.8 ± 1	^32^See Supplementary Information S2 and Table S1 for calculation of SD
**Mortality rates**			
0–3 years (± SD)	39 ± 11%	61 ± 11%	See “Results” and SI
> 3 years (adult) (± SD)	11 ± 6%	33 ± 6%	See “Results” and SI
**Dispersal**			
Proportion of gated population emigrating to ungated population	4% (Juveniles only)		Estimated from Dubey et al.^41^
Survival	39%		Juvenile survival of ungated population, see “Results”
**Initial population size**			
*n*	600	600	Webb et al.^31^ estimate a population of 595 (95% CI 389–808) for our gated population
**Carrying capacity**			
K	800	800	Estimated upper confidence interval of the population estimate from^31^

Population growth rate is central to our ability to predict population dynamics^45^ and is essentially governed by rates of fecundity, survival, immigration and emigration. Our ability to estimate fecundity and survival is relatively robust due to the long-term demographic data available for these populations. Although we cannot differentiate between mortality and permanent emigration, adult broad-headed snakes show site fidelity, and are often recaptured underneath the same rocks where they were originally captured^33,46^. Furthermore, there is virtually no suitable habitat outside of the national park into which snakes can permanently emigrate^35,47^. Population genetics for our study area showed that dispersal occurred in the juvenile life stage and was unidirectional, with juvenile dispersal movements occurring only from the gated population into the ungated population^41^. Thus, we have reasonable justification to treat the populations as closed.

### Population trajectories using population viability analysis

**Figure 1:**
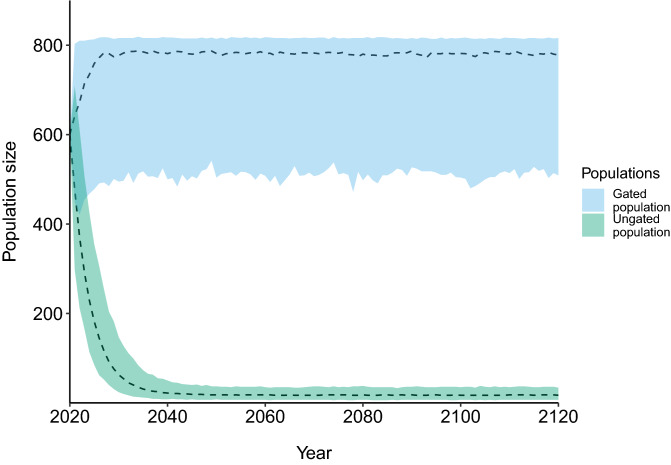
Population viability analysis for broad-headed snakes in Morton National Park, New South Wales, Australia. This figure represents the predicted population trajectories based on the life history parameters estimated from a long-term mark–recapture study of these populations. The ungated population (green) is only saved from local extinction by migration of juveniles from the gated population.

To determine the effects of illegal collecting on the ungated population, we used the program Vortex 10.2.5.0^48^ to conduct 100-year baseline scenario population viability analyses (PVA), using 1000 iterations to obtain a mean population growth rate and a probability of population extinction. The individual-based modelling approach of the Vortex software was considered suitable for this study as it provides a flexible approach to varying individual traits and we had access to detailed life history data for the species at our study sites. The baseline scenario (Fig. 1) uses our best estimates for life history parameters, all of which are either estimated in this study (see Table 1; “Results”) or are taken from previously published results from these populations of broad-headed snakes (Table 1). Where we were uncertain about any life history parameters, we employed subsequent sensitivity tests that artificially varied the parameters to determine their effect on population growth rate (see below; Table 2).

**Table 2:** Parameters varied for sensitivity testing of population viability analysis of broad-headed snake populations in Morton National Park, New South Wales, Australia.

Parameters	Mean	Minimum	Maximum
Initial population size (n)	649.8	100	1200
Carrying capacity (K)	899.6	600	1200
Harvest rate of adult females	15.5	1	30
Harvest rate of adult males	15.5	1	30
Immigration	4%	0%	8%

Using our mark–recapture data we calculated the age-specific survival rates of each population (see “Results”) and found that the annual survival of our ungated population was substantially lower than that of our gated population for both juveniles and adults (Table 1). These survival rates, however, included the impacts of illegal collecting activities. To investigate how collecting affects population viability, we made the assumption that without this impact both populations would have the same survival rates as the gated population. Therefore, for all subsequent analyses, we assumed all life history parameters to be equal between both ungated and gated populations, with unidirectional dispersal from the gated population into the ungated population.

### Sensitivity analysis

Whilst useful for predicting population trajectories^49^, PVA does not provide quantitative information about which parameters most influence population trajectories through time, nor does it account for uncertainties in estimated life history parameters^50^. To account for these uncertainties and directly assess the influence of different parameters, we artificially varied the values of five parameters for sensitivity testing in Vortex, including: (1) immigration; (2) initial population size (*n*); (3) carrying capacity (*k*); (4) harvest rate of adult females; and (5) harvest rate of adult males (Table 2). All parameters of the gated population were maintained at base scenario levels for sensitivity analysis.

Since we were interested in the relative importance of each parameter on the population growth rate, we limited variation around the parameter to values that we as experts considered biologically plausible. Unidirectional dispersal of juvenile snakes (1–3 years) from the gated population to the ungated population was estimated to be at a rate of ~ 4% of the juvenile population of origin (Table 1^41^), and this value was allowed to vary between 0 and 8%. The size of our gated population had been previous estimated as 595 (95% CI 389–808) snakes^31^, and since the available habitat of both ridges is similar in extent, we allowed initial ungated population to vary between 100 and 1200 snakes. We assumed the lowest possible carrying capacity for both populations to be close to the estimated initial population size and allowed the carrying capacity of the ungated population to double (*n* = 600–1200). Since collecting of broad-headed snakes is illegal (*Environmental Protection and Biodiversity Conservation Act 1999*), we have little knowledge about how many snakes are removed each year. We do, however, know the difference in survival between the gated and ungated populations. Since annual adult survival in the ungated population is about ~ 22% lower than that of the gated population, we can estimate that about 22% of adults (*n* ≈ 48) at the initial total population size (*n* = 600) are being removed from the population, or at least are not persisting in the population, each year. Given this uncertainty, to investigate the effect of collecting on population viability, we varied the rate of harvesting of male and female adults between 1 and 30 individuals, respectively (Table 2). In each iteration of the sensitivity analyses, stochastic variation was maintained at the same level as the base scenario. To optimise sampling of the available parameter space for each parameter, we ran 250 sensitivity samples in Vortex using the Latin hypercube sampling method.

For analysis, the summary output file from the sensitivity test in Vortex was read into the statistical program R version 4.0.2^51^. To allow for comparisons between the effects of each parameter on the stochastic population growth rate, all predictor variables were scaled and centred. To ensure there was limited correlation between the artificially manipulated predictor variables, we created pairwise scatterplots and calculated correlation coefficients using the *pairs.panels* function of the ‘psych’ package^52^.

A generalised linear model was constructed with the stochastic population growth rate as the response variable and all artificially varied parameters as predictor variables. Model predictions for the population growth rate were plotted against the main effects to visualise the relative slopes of the predictor variables. The *anova* function was used on the model object to produce an analysis of deviance table, and the percentage of deviance explained by each of the predictor variables was recorded.

### Effects of illegal collecting on population viability

To investigate and visualise how illegal collecting may be affecting the population viability of the ungated population, we maintained the assumption that without the impact of collecting both populations would have the survival rates of the gated population. To investigate whether this was a sound assumption, we ran a second PVA, where survival rates were kept at the observed levels for the gated population and harvesting of adult females (*n* = 22; or 22% of reproductive females at an initial population of 600 snakes) was imposed on the ungated population. We also included unidirectional dispersal from the gated population into the ungated population.

### Ethics approval

Animal ethics approval was granted by University of Technology Sydney Animal Ethics Committee.

## Results

### Impacts of illegal collection of snakes from the ungated population

Survival analyses in program MARK provided strong support that survival rates ($$S$$) differed between the gated and ungated populations (Table 3). In all three well-supported models (with delta AIC < 2), survival rates were higher for the gated population. In model $$S (group) p (time)$$, annual survival rates were 0.89 (± 0.05) for adults and 0.57 (± 0.08) juveniles in the gated population, and 0.67 (± 0.11 SE) for adults and 0.39 (± 0.16) juveniles in the ungated population. From model $$S \left(gated vs ungated\right) p \left(time\right),$$ which had equivalent support from the data, annual survival rates were 0.84 (± 0.05) for the gated population and 0.55 (± 0.08) for the ungated population. From the third model $$S \left(group\right) p \left(constant\right),$$ survival rates were 0.83 (± 0.04) for adults and 0.55 (± 0.08) for juveniles in the gated population, and 0.63 (± 0.11 SE) for adults and 0.39 (± 0.16) for juveniles in the ungated population, with a constant recapture rate (0.16 ± 0.03). None of the other models that we tested had any support from the data (Table 3).

**Table 3:** Results of Cormack–Jolly–Seber analyses in MARK that was used to model rates of survival (S) and recapture (p) of broad-headed snakes. The best supported models (with delta AIC < 2) are in bold font.

Model	AICc	Delta AIC	AIC weight	Model likelihood	N	Deviance
${\varvec{S}}\boldsymbol{ }({\varvec{g}}{\varvec{r}}{\varvec{o}}{\varvec{u}}{\varvec{p}})\boldsymbol{ }{\varvec{p}}\boldsymbol{ }({\varvec{t}}{\varvec{i}}{\varvec{m}}{\varvec{e}})$	**455.92**	**0.00**	**0.38**	**1.00**	**16**	**203.39**
${\varvec{S}}\boldsymbol{ }({\varvec{g}}{\varvec{a}}{\varvec{t}}{\varvec{e}}\boldsymbol{ }{\varvec{v}}{\varvec{s}}\boldsymbol{ }{\varvec{u}}{\varvec{n}}{\varvec{g}}{\varvec{a}}{\varvec{t}}{\varvec{e}}{\varvec{d}})\boldsymbol{ }{\varvec{p}}\boldsymbol{ }({\varvec{t}}{\varvec{i}}{\varvec{m}}{\varvec{e}})$	**456.02**	**0.10**	**0.36**	**0.95**	**14**	**208.00**
${\varvec{S}}\boldsymbol{ }({\varvec{g}}{\varvec{r}}{\varvec{o}}{\varvec{u}}{\varvec{p}})\boldsymbol{ }{\varvec{p}}\boldsymbol{ }({\varvec{c}}{\varvec{o}}{\varvec{n}}{\varvec{s}}{\varvec{t}}{\varvec{a}}{\varvec{n}}{\varvec{t}})$	**457.33**	**1.40**	**0.19**	**0.50**	**5**	**228.75**
${\varvec{S}}\boldsymbol{ }({\varvec{g}}{\varvec{r}}{\varvec{o}}{\varvec{u}}{\varvec{p}})\boldsymbol{ }{\varvec{p}}\boldsymbol{ }({\varvec{g}}{\varvec{r}}{\varvec{o}}{\varvec{u}}{\varvec{p}})$	459.03	3.11	0.08	0.21	8	224.12
${\varvec{S}}\boldsymbol{ }({\varvec{c}}{\varvec{o}}{\varvec{n}}{\varvec{s}}{\varvec{t}}{\varvec{a}}{\varvec{n}}{\varvec{t}})\boldsymbol{ }{\varvec{p}}\boldsymbol{ }({\varvec{t}}{\varvec{i}}{\varvec{m}}{\varvec{e}})$	470.37	14.45	0.00	0.00	13	224.58
${\varvec{S}}\boldsymbol{ }({\varvec{c}}{\varvec{o}}{\varvec{n}}{\varvec{s}}{\varvec{t}}{\varvec{a}}{\varvec{n}}{\varvec{t}})\boldsymbol{ }{\varvec{p}}\boldsymbol{ }({\varvec{g}}{\varvec{r}}{\varvec{o}}{\varvec{u}}{\varvec{p}})$	471.43	15.51	0.00	0.00	5	242.85
${\varvec{S}}\boldsymbol{ }({\varvec{c}}{\varvec{o}}{\varvec{n}}{\varvec{s}}{\varvec{t}}{\varvec{a}}{\varvec{n}}{\varvec{t}})\boldsymbol{ }{\varvec{p}}\boldsymbol{ }({\varvec{c}}{\varvec{o}}{\varvec{n}}{\varvec{s}}{\varvec{t}}{\varvec{a}}{\varvec{n}}{\varvec{t}})$	472.12	16.20	0.00	0.00	2	249.73
${\varvec{S}}\boldsymbol{ }({\varvec{t}}{\varvec{i}}{\varvec{m}}{\varvec{e}})\boldsymbol{ }{\varvec{p}}\boldsymbol{ }({\varvec{t}}{\varvec{i}}{\varvec{m}}{\varvec{e}})$	478.15	22.22	0.00	0.00	16	225.61

Each snake was assigned to one of four groups depending on its size at first capture (sub-adults and adults, or juveniles) and population (gated or ungated). Table shows AIC values and associated AIC weights, model likelihood, number of parameters (N), and model deviance.

### Baseline population viability analysis

The results of the baseline PVA showed that the gated population has a positive growth rate (*r* = 0.084) and rapidly grows to the carrying capacity which we imposed upon it (Fig. 1). This may suggest that the carrying capacity of this population is actually likely to be closer to our estimated initial population. The ungated population, however, has a negative growth rate (*r* = − 0.246) and only avoids population extinction because it is sustained by the small amount of juvenile dispersal from the gated population (Fig. 1).

### Sensitivity analysis

**Figure 2:**
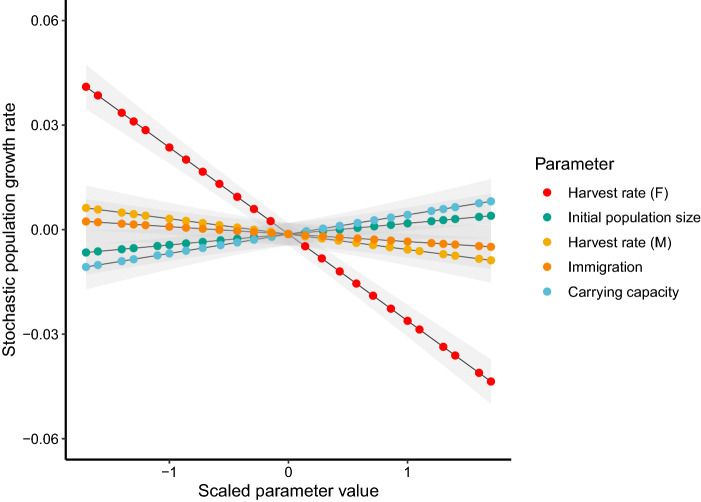
Effects that five vital rate parameters have on the stochastic population growth rate of broad-headed snakes in an ungated population suspected to be subjected to illegal collecting on the boundary of 29 Morton Nation Park, New South Wales, Australia. Parameter values have been scaled and centred to allow for comparison. Grey shading represents 95% confidence intervals.

**Figure 3:**
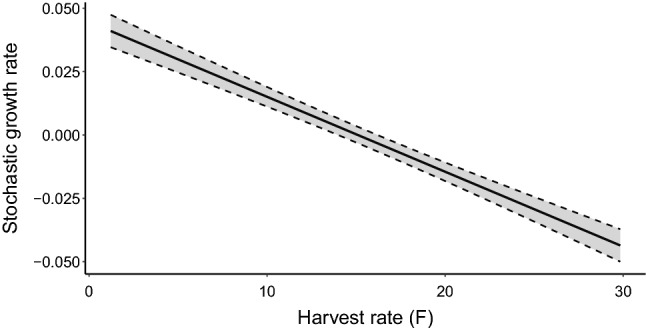
Effect that illegal collection of female broad-headed snakes has on the stochastic growth rate of a population suspected to be impacted by collecting on the boundary of Morton National Park, New South Wales, Australia.

Sensitivity analyses showed that three of the five factors we varied had a significant effect on stochastic growth rate. Carrying capacity (*k*; *X*^2^(1) = 11.26; *p* < 0.001) and harvest rate of adult males (*X*^2^(1) = 7.17; *p* < 0.01) significantly modified the stochastic growth rate, but both accounted for < 5% of the total deviance explained by the model (Table 4). Harvest rate of adult females, however, had a large effect on the stochastic growth rate (*X*^2^(1) = 226.92; *p* < 0.0001) and accounted for 91% of total deviance explained by the model (Table 4). Importantly, the analysis showed that populations were extremely sensitive to even small changes in adult female survival (Fig. 2). When all other parameters were set to their mean values (Table 2), harvest of greater than ~ 15 females per year resulted in a negative stochastic growth rate (Fig. 3).

**Table 4:** Percentage of deviance explained by each predictor variable in generalised linear models where population growth rate was the response variable for broad-headed snake populations in Morton National Park, New South Wales, Australia. Emboldened percentage deviance identifies harvest rate of females as explaining the majority of deviance in the model and having the largest effect on stochastic growth rate.

	Df	Deviance	Residual df	Residual deviance	Percentage of deviance
NULL	NA	NA	250	0.339	NA
Harvest rate of females	1	0.158	249	0.181	**91.06**
Carrying capacity (K)	1	0.007	247	0.171	4.28
Harvest rate of males	1	0.005	246	0.167	2.560
Initial population size (n)	1	0.002	248	0.179	1.41
Immigration	1	0.001	245	0.166	0.65

### Effects of illegal collecting on population viability

**Figure 4:**
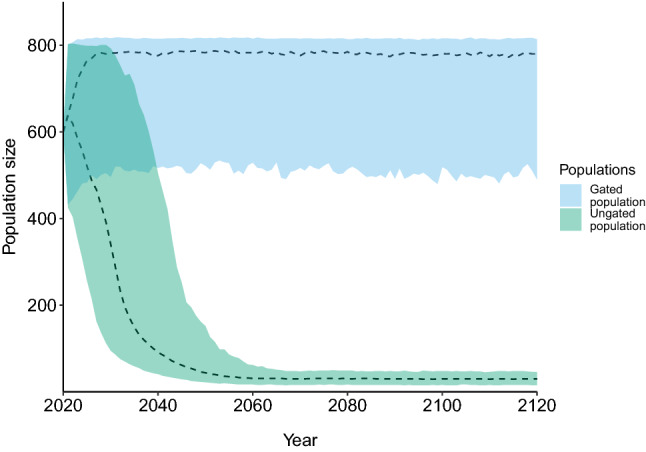
Population viability analysis for broad-headed snakes in Morton National Park, New South Wales, Australia. This figure represents simulated population trajectories where survival estimates for the gated population was assumed for both populations, but a harvesting rate of 22 females per year was imposed on the ungated population. All other life history parameters were maintained. The ungated population (green) is only saved from local extinction by dispersal of juveniles from the gated population.

When we ran a PVA using the same population parameters (particularly survival rates) for each population, with unidirectional dispersal (Table 1), but incorporated a harvest rate of 22 adult females per year in the ungated population, we observed that, similarly to the baseline PVA, the ungated population has a negative growth rate (*r* = − 0.025) and only avoids population extinction because it is sustained by the small amount of juvenile dispersal from the gated population (Fig. 4).

## Discussion

Here, we provide one of the first attempts to model the impact of illegal collecting on a population of threatened reptiles. Our results provide strong evidence that human collection of endangered broad-headed snakes from wild populations to supply the illegal pet trade can drive rapid population declines. Over the 12-year period from 2007 to 2019, humans disturbed rock outcrops on the ungated plateau every year, and they disturbed more ungated sites than gated sites^37^ (see Supplementary Information). Consistent with the hypothesis that humans removed snakes from the ungated population, we found that annual survival rates of broad-headed snakes were ~ 22% lower in the ungated population than the gated population. PVA using the life history parameters of each population show that the ungated population is on a trajectory towards local extinction, presumably due to the impacts of illegal collecting for the wildlife trade.

Our sensitivity analyses showed that increases in adult female mortality rates had the most significant effect on population growth rates of broad-headed snakes, consistent with the results of demographic studies on other long-lived species (e.g.^53,54^). Surprisingly, without dispersal, the removal of less than 20 adult females per year rapidly drove the ungated population to local extinction. While few studies have modelled the effects of collecting on population growth, other studies on long-lived vertebrates have shown that small increases in mortality rates can have similar effects. For example, in a population of the Egyptian vulture (*Neophron percnopterus*), small increases in adult mortality from wind turbine collisions decreased population sizes and time to extinction^55^. Similarly, the current levels of illegal collecting of broad-headed snakes are not sustainable. During annual surveys of the ungated population, we found 0.46 adult female broad-headed snakes per hour of survey effort (Webb unpubl. data). It would, therefore, take approximately 5 days for a team of two people to remove 20 adult females from this population. Because broad-headed snakes are easy to locate on these plateaux during the cooler months, it would not be difficult for collectors to remove a high proportion of females from this population. Studies on other reptiles show that even a single collecting event can dramatically impact small populations. For example, in February 2010, three collectors removed almost half of the breeding population of jewelled geckos (*Naultinus gemmeus*) from a small population in New Zealand^56–58^.

Our viability analyses suggest that the only factor rescuing the ungated population from imminent extinction is immigration of juveniles from the adjacent and better protected gated population. However, the model ignores habitat quality, which is also an important determinant of population viability^59^. Numerous studies have shown that all else being equal, populations are more likely to persist in patches of higher quality habitat^60–62^. With the exception of our study sites, which were restored with artificial rocks^63,64^, the habitat quality of rock outcrops on the ungated plateau is lower than on rock outcrops on the gated plateau^37^. Over the last three decades, bush rock collectors have removed sandstone rocks from rock outcrops on the ungated plateau to supply the landscape garden industry^65^. Most rock outcrops on the ungated plateau are accessible via fire trails, and consequently, many outcrops have been stripped of their natural rocks^30,37^. The rocks stolen by rock thieves are similar in size to those selected by broad-headed snakes, and the velvet gecko *Amalosia lesueurii*^65^, which forms a large component of the diet of juvenile broad-headed snakes. The removal of surface rocks will decrease prey abundance^65^ and the availability of thermally suitable shelter sites, which may render juvenile snakes more vulnerable to predation from birds or sympatric small-eyed snakes^66^. Thus, poor habitat quality may contribute to lower survival rates of immigrants, so that the rescue effect of immigration may be negligible^67^.

There are a number of biological attributes that are thought to predispose a species to risk of extinction. Although some species, including some reptiles (e.g.^24^), may be able to sustain reasonably high levels of harvesting from the wild without suffering negative growth rates^68^, some species cannot sustain even low levels of wild harvesting. Factors such has high trophic level, low population density, slow life history, small geographical range size and ecological specialisation are all significantly associated with high extinction risk^27,54^, and may make some species exceptionally vulnerable to the impacts of the wildlife trade. To date, most studies on broad-headed snakes have focused on their ecological specialisation (i.e. dependence on sandstone rocks) as the cause of their engendered status (e.g.^31,65,69^). Our results underscore how a slow life history can render a species vulnerable to even low levels of illegal collecting, even in the absence of other potential threats, such as increases in predation by invasive predators (e.g. cats, foxes), or high intensity wildfires that are predicted to increase in frequency in future^36,70^.

We acknowledge that the lower rates of survival we detected in the ungated population could be caused by humans removing snakes, and also, by humans breaking and disturbing rocks whilst searching for snakes. Broad-headed snakes are spectacularly coloured (see Fig. S1), and so are sought after by herpetologists and photographers who, by necessity, have to lift rocks to find the snakes. In many cases, these enthusiasts often leave rocks overturned or displaced from their original locations. Previous studies have shown that displacement of rocks (i.e. not returning them to their original locations) changes their crevice size and thermal profiles, making them unsuitable for broad-headed snakes and their prey^59^. Potentially, such subtle changes to habitat quality could also affect survival rates of snakes that are not collected.

Globally, we face a rapidly accelerating extinction crisis. In many cases, we lack the detailed understanding of species’ life histories and ecologies required to determine what is causing their extirpation. Our study highlights the value of detailed long-term demographic studies not only for identifying threats to populations, but for modelling the impacts of such threats on population viability. We also show that management actions aimed at deterring illegal collectors and restricting their access to sensitive populations can help reduce those threats. In the current example, the erection of locked gates and installation of security cameras has helped reduce disturbance to the gated population, and this population is not at imminent risk of local extinction. By contrast, the ungated population is on a trajectory to extinction, and urgent measures are necessary to reverse this decline. The installation of locked gates and cameras, and restoration of degraded habitats, may well help reverse the current decline, but tougher penalties are also required for poachers caught collecting or selling broad-headed snakes. If we can help authorities to enact such actions, we may yet be able to prevent an evolutionarily significant population of endangered species from going extinct.

## Supplementary Information

Supplementary Information

## Data Availability

All data available upon request from authors.

## Code availability

All code available upon request from authors.
