# Supplementary material for: Slow life history leaves endangered snake vulnerable to illegal collecting
Source: Sci Rep. 2021 Mar 8;11:5380. doi: 10.1038/s41598-021-84745-1 (PMC7970890; doi:10.1038/s41598-021-84745-1)
Supplement: Supplementary file 1 — Supplementary Information [file 41598_2021_84745_MOESM1_ESM.docx]

**Slow life history leaves endangered snake vulnerable to illegal collecting**

CHRIS J. JOLLY, BRENTON VON TAKACH & JONATHAN K. WEBB

**Electronic Supplementary Information**

**Materials and methods**

**Study species**

The broad-headed snake (*Hoplocephalus bungaroides*) is a small (to 90 cm long) brightly coloured nocturnal elapid snake (Fig. S1) that is entirely restricted to sandstone habitats within 200 km of the city of Sydney^1^. Broad-headed snakes require sun-exposed rocks and crevices for thermoregulation, and juveniles feed heavily on lizards, including the velvet gecko^2^, a species that also requires sun-exposed stones for thermoregulatory sites^3,4^. Loss of habitat from urbanisation, and removal of ‘bush rocks’ has contributed to the decline of broad-headed snakes and velvet geckos^5^. During the cooler months, broad-headed snakes occur on rock outcrops where they thermoregulate under sun exposed rocks. In summer, when rocks are too hot for snakes to tolerate, adults move away from rock outcrops to adjacent forests where they shelter inside tree hollows^3,6,7^. In summer adults maintain home ranges of up to 10 ha (mean 3-4 ha), while ranges are much smaller (< 0.5 ha) when snakes occupy rock outcrops ^7,8^. Females display site fidelity, often returning to the same rock outcrops each year, where they occupy rocks used in previous years ^8^.

**
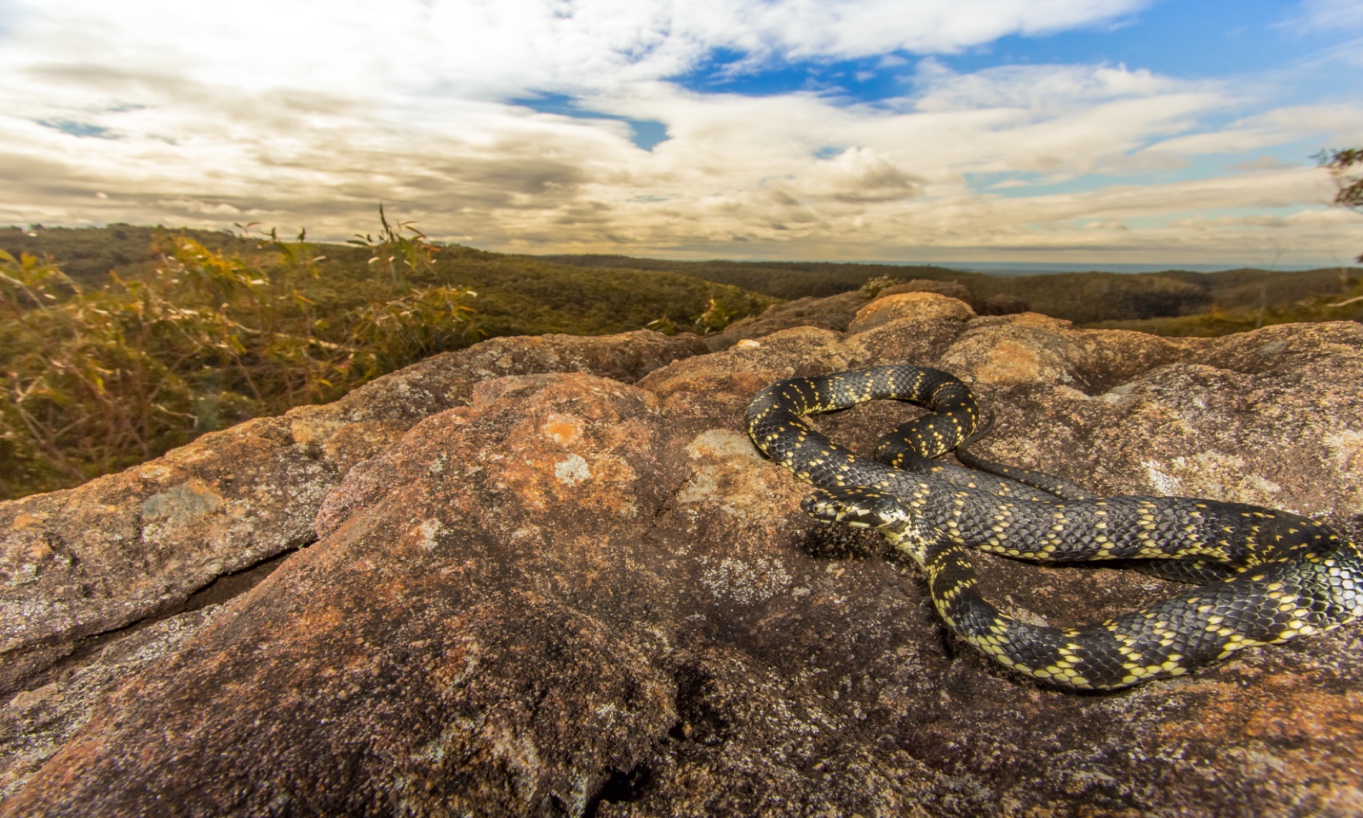
**

**Figure S1.** Broad-headed snake (*Hoplocephalus bungaroides*) on a sandstone plateau in New South Wales, Australia (Photo: Chris Jolly).

**Collection of broad-headed snakes by hobbyists**

The collection of broad-headed snakes from the wild has long been suggested as a causal factor that has contributed to the species decline throughout its geographic range^9,10^. Broad-headed snakes were kept in large numbers by collectors during the 1970’s, when no licence was required to hold snakes. At several known collecting sites near Sydney, where broad-headed snakes were once abundant in the 1970’s, the species is now rare or absent^11^.

In NSW, the National Parks and Wildlife Service enacted a wildlife amnesty in 1997, whereby reptile keepers could obtain licences to keep any native reptiles, including broad-headed snakes. This wildlife amnesty coincided with a significant disturbance to rocks on rock outcrops in Morton National Park that was accompanied by a significant decline in broad-headed snake survival and abundance. Notably, this collection event did not affect survival rates of the eastern small-eyed snake (*Cryptophis nigrescens*) (Fig. S2), a common species not favoured by hobbyists, suggesting that collectors had indeed targeted and removed broad-headed snakes from the population^12^.

Currently, individuals can legally keep broad-headed snakes in captivity in NSW, provided that the snakes were purchased from a licenced keeper or were declared during the 1997 amnesty^13^. However, until recently, keepers were not required to microchip captive broad-headed snakes, nor were DNA samples taken from those individuals^14^. This meant there was no way to know whether snakes purchased from licenced keepers were captive born or wild-caught individuals. In 2010, to address this concern, wildlife authorities worked with geneticists to sequence DNA from private collections and wild animals. The genetic results were quite striking—there was a high degree of genetic similarity between individuals from captive collections and wild populations, but not between zoo held animals and wild populations, suggesting that there was extensive movement of wild animals into private collections. Perhaps not surprisingly, after keepers were required to microchip broad-headed snakes and provide DNA samples to wildlife authorities, demand for licences to keep broad-headed snakes declined markedly^13^.

While we have no data on the numbers of broad-headed snakes traded on the black market, the species is rare and spectacularly coloured (Fig. S1), and so continues to be highly sought after by photographers, herpetologists, and snake enthusiasts. The high demand for the species is evidenced by extensive habitat disturbance to sites where the species is known to occur^11,15^, and at sites following the uploading of broad-headed snake records on the BIONET Atlas (<http://www.bionet.nsw.gov.au/>). For example, in areas near Morton National Park, NSW, National Parks and Wildlife Services rangers found rocks crowbarred off cliffs, overturned, and displaced at several localities after broad-headed snake records were uploaded to BIONET (Phil Craven, NSW NPWS, pers. comm.). This led to Department of Planning Industry and Environment ‘denaturing’ BIONET records for broad-headed snakes so that they no longer gave accurate location data.

**
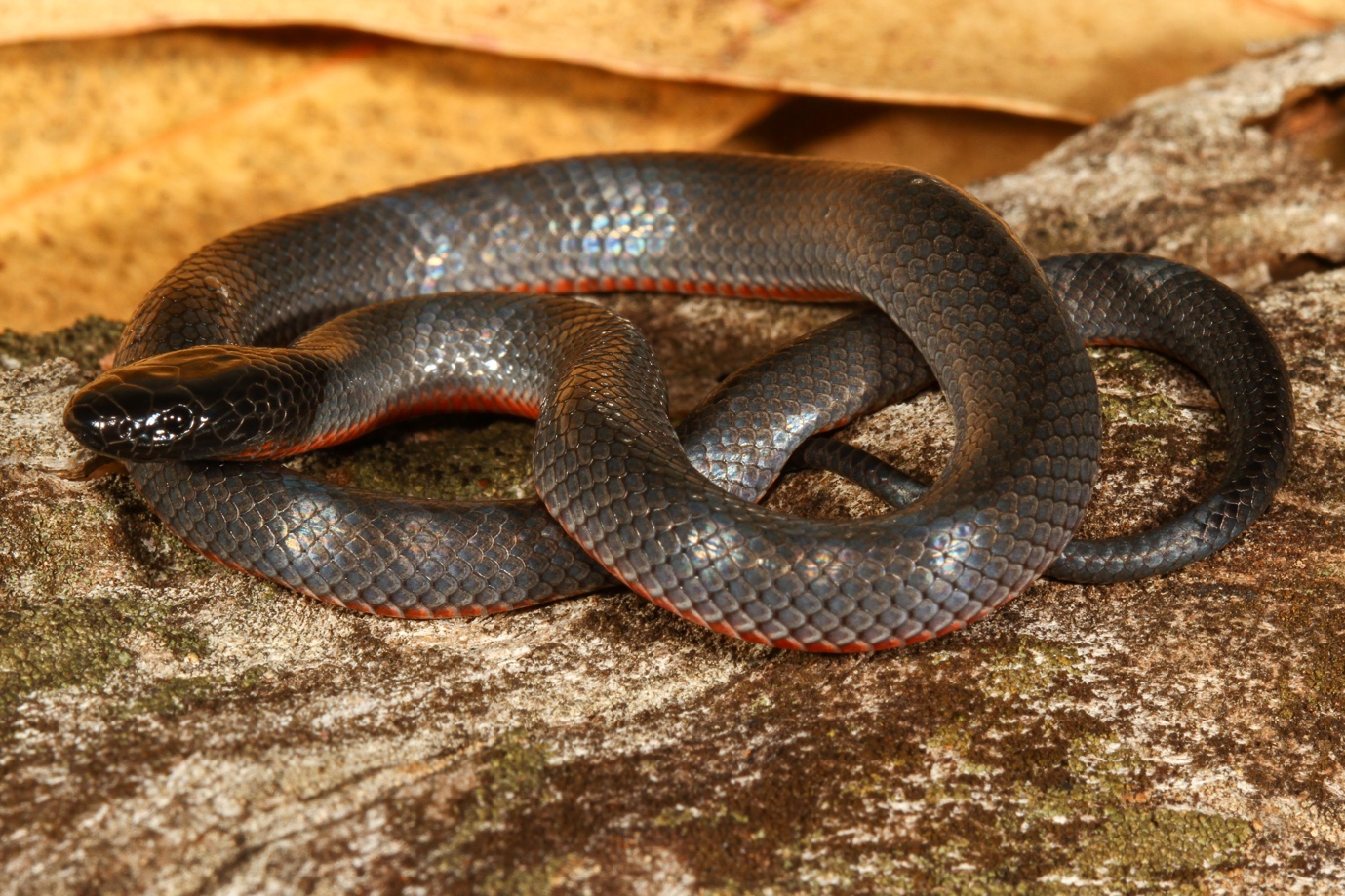
**

**Figure S2.** Eastern small-eyed snake (*Crytophis nigrescens*), New South Wales, Australia (Photo: Chris Jolly).

**Estimation of life history parameters from study population**

Because good estimates of parameter uncertainty are necessary to construct informative stochastic demographic models, we used program MARK to obtain estimates of environmental (process) variation around survival rates. We did this using the variance components subroutine in MARK (appendix D, MARK Book v 19, see^16^). For this analysis, we used the mark–recapture data set for the gated population, with two groups (juveniles and adults). We then ran CJS models to determine the best supported model from the data. The data showed some over dispersion (bootstrap GOF test, p = 0.05, 1000 replicates) so we used c-hat = 1.27 to adjust our candidate model set in MARK. From this analysis, the best supported model was $S (group) p (time)$, indicating that survival rates differed between adults and juveniles (Table S1). We then used the variance components subroutine in program MARK to estimate annual variation around survival rates for each age class separately. For this analysis we used the model $S (group x time) p (constant)$. This yielded estimates of annual survival for adults and juveniles of 0.89 and 0.61 respectively. The standard deviations for these estimates were calculated from the estimates of sigma, and were 0.057 and 0.108 for adults and juveniles respectively.

**Table S1.** Results of Cormack–Jolly–Seber analyses in MARK that was used to model rates of survival (S) and recapture (p) of broad-headed snakes from the gated population. Each snake was assigned to one of two groups depending on its size at first capture (sub-adults and adults, or juveniles).Table shows AIC values and associated AIC weights, model likelihood, number of parameters (N), and model deviance. The best-supported model is shown in bold font.

| Model | AICc | Delta  AICc | AICc  weight | Model Likelihood | N | Deviance |
| --- | --- | --- | --- | --- | --- | --- |
| $\boldsymbol{S (group) p (time)}$ | **297.9946** | **0.00** | **0.59** | **1.00** | **14** | **122.06** |
| $\boldsymbol{S (group) p (constant)}$ | 299.6107 | 1.62 | 0.27 | 0.45 | 3 | 147.76 |
| $\boldsymbol{S (group) p (group)}$ | 301.2301 | 3.24 | 0.12 | 0.20 | 4 | 147.30 |
| $\boldsymbol{S (constant) p (group)}$ | 306.0114 | 8.02 | 0.01 | 0.02 | 3 | 154.17 |
| $\boldsymbol{S (constant) p (time)}$ | 306.5289 | 8.53 | 0.01 | 0.01 | 13 | 132.90 |
| $\boldsymbol{S (constant) p (constant)}$ | 308.5055 | 10.51 | 0.00 | 0.01 | 2 | 158.72 |
| $\boldsymbol{S (time) p (group)}$ | 317.2972 | 19.30 | 0.00 | 0.00 | 14 | 141.36 |
| $\boldsymbol{S (time) p (constant)}$ | 319.8637 | 21.87 | 0.00 | 0.00 | 13 | 146.23 |
| $\boldsymbol{S (group) p (group x time)}$ | 320.6342 | 22.64 | 0.00 | 0.00 | 26 | 115.02 |
| $\boldsymbol{S (time) p (time)}$ | 325.4935 | 27.50 | 0.00 | 0.00 | 23 | 127.67 |
| $\boldsymbol{S (constant) p (group x time)}$ | 325.5252 | 27.53 | 0.00 | 0.00 | 25 | 122.54 |
| $\boldsymbol{S (group x time) p (constant)}$ | 332.3954 | 34.40 | 0.00 | 0.00 | 25 | 129.41 |

**References**

1. Cogger, H. G. *Reptiles & amphibians of Australia*. (CSIRO Publishing, 2018).

2. Webb, J. K. & Shine, R. Ecological characteristics of a threatened snake species, *Hoplocephalus bungaroides* (Serpentes, Elapidae). *Anim Conserv* **1**, 185–193 (1998).

3. Webb, J. K. & Shine, R. Using thermal ecology to predict retreat-site selection by an endangered snake species. *Biol Conserv* **86**, 233–242 (1998).

4. Webb, J. K. & Shine, R. Thermoregulation by a Nocturnal Elapid Snake (*Hoplocephalus bungaroides*) in Southeastern Australia. *Physiol. Zool.* **71**, 680–692 (1998).

5. Shine, R., Webb, J. K., Fitzgerald, M. & Sumner, J. The impact of bush-rock removal on an endangered snake species, *Hoplocephalus bungaroides* (Serpentes: Elapidae). *Wildl. Res.* **25**, 285 (1998).

6. Webb, J. K. & Shine, R. Out on a limb: Conservation implications of tree-hollow use by a threatened snake species (*Hoplocephalus bungaroides*: Serpentes, Elapidae). *Biol Conserv* **81**, 21–33 (1997).

7. Croak, B. M., Crowther, M. S., Webb, J. K. & Shine, R. Movements and Habitat Use of an Endangered Snake, *Hoplocephalus bungaroides* (Elapidae): Implications for Conservation. *PLoS ONE* **8**, e61711 (2013).

8. Webb, J. K. & Shine, R. A field study of spatial ecology and movements of a threatened snake species, *Hoplocephalus bungaroides*. *Biol Conserv* **82**, 203–217 (1997).

9. Hersey, F. Broad-headed snake *Hoplocephalus bungaroides*. in *Endangered animals of New South Wales.* 38–40 (National Parks and Wildlife Service, Sydney., 1980).

10. Burbidge, A. A. & Jenkins, R. W. G. *Endangered vertebrates of Australia and its island territories.* (Australian National Parks and Wildlife Service., 1984).

11. Newell, D. & Goldingay, R. Distribution and habitat assessment of the Broad-headed Snake *Hoplocephalus bungaroides*. *Aust Zool* **33**, 168–179 (2005).

12. Webb, J. K., Brook, B. W. & Shine, R. Collectors endanger Australia’s most threatened snake, the broad-headed snake *Hoplocephalus bungaroides*. *Oryx* **36**, 170–181 (2002).

13. Hogg, C. J., Dennison, S., Frankham, G. J., Hinds, M. & Johnson, R. N. Stopping the spin cycle: genetics and bio-banking as a tool for addressing the laundering of illegally caught wildlife as ‘captive-bred’. *Conserv. Genet. Resour.* **10**, 237–246 (2018).

14. Frankham, G. J., Hinds, M. C. & Johnson, R. N. Development of 16 forensically informative microsatellite loci to detect the illegal trade of broad headed snakes (*Hoplocephalus bungaroides*). *Conserv. Genet. Resour.* **7**, 533–535 (2015).

15. Goldingay, R. Between a rock and a hard place: conserving the broad-headed snake in Australia’s oldest National Park. *Proc. Linn. Soc. NSW* **120**, 1–10 (1998).

16. Cooch, E. & White, G. *Using MARK—a gentle introduction.* (Cornell University, 2001).
